# Editorial: Endocrine Diseases of Newborn: Epidemiology, Pathogenesis, Therapeutic Options and Outcome

**DOI:** 10.3389/fped.2021.764710

**Published:** 2021-09-23

**Authors:** Paula Caroline Midgley, Amanda Lesley Ogilvy-Stuart

**Affiliations:** ^1^Department of Child Life and Health, University of Edinburgh, Edinburgh, United Kingdom; ^2^Neonatal Unit, Rosie Hospital, Cambridge University Hospitals NHS Foundation Trust, Cambridge, United Kingdom

**Keywords:** glucose homeostasis, calcium homeostasis, hypopituitarism, adrenal insufficiency, differences in sex development, minipuberty, congenital adrenal hyperplasia, neonatal bone disorders

The aim of this collection is to provide information on recent advances and current thinking in selected areas of Neonatal Endocrinology, as we believe that this area can be challenging in presentation and management for both neonatologists and pediatric endocrinologists. To this end, we invited several leaders in the field to contribute. In addition, we have included 3 manuscripts submitted to Frontiers that fell within the remit of our topic.

The collection focuses on selected conditions presenting in the newborn consisting of a series of 10 mini-reviews, including some which may be considered the “go to” article for up-to-date science and management of various conditions.

The neonatal period is complicated by unique physiology, because of the transition from intrauterine life including the influence of maternal and placental hormones, and “normal” values for hormone levels can be difficult to define, and many change with increasing postnatal age. The measurement of hormone levels is further complicated by numerous additional circulating steroids which may interfere with assays, and the challenges of small sample size. In the context of this milieu the collection of articles ranges from common neonatal problems such as hypoglycemia to rare disorders such as neonatal bone disease. The content of all the articles in the collection is summarized below.

Bosch i Ara et al. provide an extensive review of congenital hypopituitarism, with a thorough review of the science, including the known genetics, phenotype-genotype correlations, syndromic, and non-syndromic hypopituitarism with helpful accompanying tables. They also provide a comprehensive guide to the clinical presentation including red-flag symptoms and signs, diagnosis, assessment and management of pituitary hormone deficiencies.

Di Dalmazi et al. present a study on the effect of maternal and neonatal factors on neonatal TSH levels, using retrospective data from screening for congenital hypothyroidism in 62,132 infants in Abruzzo, Italy, a relatively iodine deficient area. As both sex and postnatal age at collection affected the TSH level, they advocate the use of locally derived TSH cut-offs for both and provide local percentile charts for TSH based on their data. Effects of maternal and neonatal factors were modest, but the study was limited by data routinely collected in the screening programme and the unknown contribution that iodine deficiency may have played in this population.

Buonocore et al. describe causes of adrenal insufficiency, with a focus on genetic conditions that present in the first few months of life. This superb overview provides clear and concise information on complex pathways and conditions, identifying seminal features of presentation in a manner that is easy to read. Testing for adrenal insufficiency was not in the remit of this invited review, but assessment of the HPA axis can be found in the review of congenital hypopituitarism by Bosch i Ara et al..

Balsamo et al. explore the breadth of conditions presenting as congenital adrenal hyperplasia in the newborn period, and provide insight into some of the rarer conditions. They describe how clinical characteristics and diagnostic tests may be used to distinguish between these, and how steroidogenic biochemistry is evolving in this field. Therapeutic approaches are also included.

Li et al. from China present a systematic review and meta-analysis of the screening results for congenital adrenal hyperplasia involving 7.85 million newborns. The incidence of 1/23,024 was higher in males than females, possibly as a result of the gender imbalance in China, the greater attention paid to male infants resulting in a higher recall rate, or the diagnosis of ambiguous genitalia before or after birth in females, making screening unnecessary in girls.

With advancement in the genetic diagnosis of disorders of sex development, Bertelloni et al. outline a different approach to the investigation of 46XY DSD to the extensive, often repeated and invasive laboratory testing, by using advanced genetic technologies (next generation sequencing, whole exome sequencing, targeted CGH array) as the first line test after karyotyping and salt-loss exclusion which may result in a molecular diagnosis and guide more targeted biochemical investigations. A causative genetic diagnosis allows for accurate prognosis and recurrence risk.

Lucaccioni et al. review the current understanding of minipuberty and using this window as an opportunity for the diagnosis, and potentially treatment, of babies with DSD which could alter the natural history. They highlight the hormonal changes that occur and how minipuberty modulates neurobehavioral development.

Edwards and Harding review clinical aspects of transient neonatal hypoglycemia. They discuss pathophysiology, controversy over definitions of hypoglycemia, when and how to make blood glucose measurements, and use of glucose gel to prevent hypoglycemia. They highlight the need for evidence as to whether transient asymptomatic hypoglycemia is associated with brain injury, and if so, at what level or duration.

Chandran et al. report a family with a novel HNF4A mutation presenting with differing phenotypic presentations of glucose dysregulation. They describe an infant with diazoxide responsive hyperinsulinaemic hypoglycemia, who shares a novel HNF4A mutation with a sister who had transient neonatal hypoglycemia, and his father who developed diabetes at the age of 15. Implications of the genetic diagnosis on treatment and prognosis are discussed.

Beardsall reviews hyperglycemia with a focus on the preterm infant, and discusses pathogenesis, glucose insensitivity and insulin resistance, relative insulin deficiency, the clinical consequences of hyperglycemia, and its clinical management. The review provides a wealth of information on underlying mechanisms, which may be of particular value for neonatologists.

Beltrand et al. describe how the recent advances in neonatal diabetes mellitus can guide management and how the known genetic mutations define the pathophysiology by causing either abnormal β-cell function or abnormal pancreatic morphology. The authors provide a detailed clinical description of both the permanent and transient forms. The challenge of insulin therapy in maintaining normoglycaemia is discussed, as is the management of those with mutations in the K_ATP_ channel with suphonylureas to which the channel remain sensitive in 90% of cases. A helpful appendix provides practical advice on switching from insulin to glibenclamide, the oral suspension of which is licensed for children in the European Union.

In the review of current insights into disorders of calcium and phosphate in the newborn, Taylor-Miller and Allgrove examine the current understanding of fetal-to-neonatal mineral homeostasis mechanisms (calcium, phosphorus, magnesium) as well as vitamin D. Recent genetic discoveries have shed light on the pathophysiology of some causes of neonatal hypo and hypercalcemia. The presentation and management of bone fragility is discussed as well as the investigation and management of disorders of calcium and phosphorus homeostasis.

Saraff et al. describe an approach to neonatal bone disorders for clinicians, highlighting that early and accurate diagnosis in these rare disorders can be important for potentially life-saving treatment. The review includes structural bone defects, and bone mineralization defects, and an approach to diagnosis and management. This practical approach could be extremely useful for neonatal clinicians.

This series of articles was conceived with Dr. Paolo Ghirri, Associate Professor of Pediatrics at the University of Pisa, where he was the director of Neonatology at the Santa Chiara. Following his sudden and untimely death during the planning stage, we decided to dedicate the series to his life and work and made our contributors aware of this at the time of invitation.

## Author Contributions

Both authors contributed equally to this editorial and approved it for publication.

## Conflict of Interest

The authors declare that the research was conducted in the absence of any commercial or financial relationships that could be construed as a potential conflict of interest .

## Publisher's Note

All claims expressed in this article are solely those of the authors and do not necessarily represent those of their affiliated organizations, or those of the publisher, the editors and the reviewers. Any product that may be evaluated in this article, or claim that may be made by its manufacturer, is not guaranteed or endorsed by the publisher.

